# A Raman Imaging Approach Using CD47 Antibody-Labeled SERS Nanoparticles for Identifying Breast Cancer and Its Potential to Guide Surgical Resection

**DOI:** 10.3390/nano8110953

**Published:** 2018-11-20

**Authors:** Ryan M. Davis, Jos L. Campbell, Sean Burkitt, Zhen Qiu, Soyoung Kang, Mana Mehraein, Dominie Miyasato, Helen Salinas, Jonathan T. C. Liu, Cristina Zavaleta

**Affiliations:** 1 Department of Radiology, Stanford University, 1201 Welch Road, Stanford, CA 94305, USA; ryan.m.davis5@gmail.com; 2 Department of Biomedical Engineering, University of Southern California, 1002 Child’s Way, Los Angeles, CA 90089, USA; joscampb@usc.edu (J.L.C.); sburkitt@usc.edu (S.B.); mehraein@usc.edu (M.M.); dmiyasat@usc.edu (D.M.); hsalinas@usc.edu (H.S.); 3 Department of Biomedical Engineering, Michigan State University, 775 Woodlot Drive, East Lansing, MI 48823, USA; qiuzhen@egr.msu.edu; 4 Department of Mechanical Engineering, University of Washington, 3900 East Stevens Way NE, Seattle, WA 98195, USA; Soyoungk@uw.edu (S.K.); jonliu@uw.edu (J.T.C.L.); 5 Department of Pathology, University of Washington, 1959 NE Pacific St, Seattle, WA 98195, USA

**Keywords:** nanomaterials, Raman mapping, bioimaging, SERS, surgical guidance

## Abstract

Raman spectroscopic imaging has shown great promise for improved cancer detection and localization with the use of tumor targeting surface enhanced Raman scattering (SERS) nanoparticles. With the ultrasensitive detection and multiplexing capabilities that SERS imaging has to offer, scientists have been investigating several clinical applications that could benefit from this unique imaging strategy. Recently, there has been a push to develop new image-guidance tools for surgical resection to help surgeons sensitively and specifically identify tumor margins in real time. We hypothesized that SERS nanoparticles (NPs) topically applied to breast cancer resection margins have the potential to provide real-time feedback on the presence of residual cancer in the resection margins during lumpectomy. Here, we explore the ability of SERS nanoparticles conjugated with a cluster of differentiation-47 (CD47) antibody to target breast cancer. CD47 is a cell surface receptor that has recently been shown to be overexpressed on several solid tumor types. The binding potential of our CD47-labeled SERS nanoparticles was assessed using fluorescence assisted cell sorting (FACS) on seven different human breast cancer cell lines, some of which were triple negative (negative expression of estrogen receptor (ER), progesterone receptor (PR), and human epidermal growth factor receptor-2 (HER2)). Xenograft mouse models were also used to assess the ability of our Raman imaging system to identify tumor from normal tissue. A ratiometric imaging strategy was used to quantify specific vs. nonspecific probe binding, resulting in improved tumor-to-background ratios. FACS analysis showed that CD47-labeled SERS nanoparticles bound to seven different breast cancer cell lines at levels 12-fold to 70-fold higher than isotype control-labeled nanoparticles (*p* < 0.01), suggesting that our CD47-targeted nanoparticles actively bind to CD47 on breast cancer cells. In a mouse xenograft model of human breast cancer, topical application of CD47-targeted nanoparticles to excised normal and cancer tissue revealed increased binding of CD47-targeted nanoparticles on tumor relative to normal adjacent tissue. The findings of this study support further investigation and suggest that SERS nanoparticles topically applied to breast cancer could guide more complete surgical resection during lumpectomy.

## 1. Introduction

Raman imaging of surface enhanced Raman scattering (SERS) nanoparticles (NPs) is an optical technique that offers unsurpassed sensitivity (on the order of fM) and multiplexing capabilities to the field of molecular imaging [1,2]. Incorporating these unique characteristics into a new diagnostic tool that surgeons can use in the operating room (OR) has the potential to significantly impact their ability to successfully identify and remove the entire tumor while minimizing damage to healthy neighboring tissue and decreasing the chances of recurrence and the need for repeat surgery.

Identifying tumor margins during surgical resection is a critical determinant of patient outcome. One of the biggest challenges faced by oncologic surgeons in the OR is determining where the tumor they are resecting begins and ends. Obtaining negative tumor margins can be essential to the patient’s survival [3]. Current intraoperative surgical guidance depends largely on visual cues and tactile feedback, which may be highly subjective and inaccurate in diseased tissues. In addition to the negative oncologic consequences, positive tumor margins may necessitate reoperation, thereby increasing associated patient morbidity and healthcare expenditure (e.g., imaging, anesthesia, pathology) [4,5,6].

There is a major unmet need for imaging technologies that can guide more complete resection of breast cancer. In the US, over 200,000 women are diagnosed with breast cancer and 40,000 women will die from the disease each year [7,8]. Approximately 75% of new breast cancer cases undergo lumpectomy, a breast-conserving surgery, which removes the cancerous tumor while preserving as much of the breast as possible [9]. Unfortunately, up to 60% of lumpectomy patients require additional surgery due to positive margins [4,5,6,10,11,12].

For breast lumpectomies, the current standard of care at most institutions relies on calcifications seen on a mammography to target and delineate the tumor mass, such that the tumor may be resected along with an arbitrary 2 cm margin beyond the presumed edge of the tumor. The excised tumor is then taken and prepared for histopathological examination. Several days later, a pathology report is compiled to reveal whether the surgeon was able to achieve negative tumor margins. If positive tumor margins are detected, the patient is called in again for re-excision of the residual tumor. In 20–60% of cases, complete tumor removal is not achieved during the first resection procedure, especially when the tumor is not perfectly round, or adjacent tumor areas are not detected on either mammography or magnetic resonance imaging (MRI) [13]. Impedance monitoring has also been used to determine if abnormal-looking areas are cancerous; however, its role in the operating room remains uncertain as it can add up to an hour to the total surgery time. Every minute in the operating room is precious, especially when a single minute can cost over $100 [14].

Our proposed imaging strategy involves the use of a Raman imaging device to detect SERS nanoparticles that will act as tumor-targeting contrast agents. Surgeons will be able to utilize the Raman imaging device in the OR to sensitively detect these tumor-targeting contrast agents and help guide tumor resection. A ratiometric imaging strategy will be implemented to quantify specific vs. nonspecific probe binding, resulting in a higher tumor-to-background ratio and improved detection of residual tumor in the OR.

In this study, we investigated the expression level of CD47 on several breast cancer cell lines to evaluate if it could be a useful breast tumor target for our SERS nanoparticle imaging strategy. CD47 is a glycoprotein expressed on the surface of many cells in the body. CD47 binds to signal regulatory protein alpha (SIRPα), which is a receptor on macrophages. When this particular ligation occurs, a signal is released to prevent phagocytosis of the cells with CD47. This signal ensures that healthy autologous cells are not inadvertently phagocytosed [15]. Although CD47 expression protects some normal cells from being phagocytosed, CD47 transcript and protein expressions are also seen, and in some cases to a higher degree, in cancer cells to protect themselves from being phagocytosed [16]. Cancerous cells, including those of acute myeloid leukemia, bladder cancer, and breast cancer, have been found to overexpress CD47 as a means to evade the immune system [15]. The increased CD47 expression on cancer cells would also increase the chance of cancer cell survival and is sometimes responsible for new tumor masses and even tumor relapse [15]. Researchers have capitalized on this trait to target tumor cells for therapy. Monoclonal antibodies are employed to bind with CD47, resulting in increased phagocytosis and even tumor death [17]. The CD47 blocking antibodies have been found to decrease tumor size and metastasis in several pre-clinical studies as well as initiate an antitumor cytotoxic T cell immune response [15,18,19,20].

As a result of its previous preclinical and clinical success for tumor targeting, we believe CD47 antibody has the potential to be labeled with our SERS nanoparticles for effective targeting of breast cancer. In this study, breast cancer cell lines with combinations of estrogen receptor (ER), progesterone receptor (PR), and human epidermal growth factor receptor (HER2) biomarkers as well as triple negative cell lines (Table 1) were interrogated for CD47 expression. Surface-enhanced Raman scattering (SERS) nanoparticles conjugated with CD47-specific antibodies were then assessed for tumor targeting efficiency in cell culture and xenograft models. Since our SERS nanoparticles are also fluorescently labeled, FACS analysis may be utilized to determine the binding levels of our CD47 antibody-labeled SERS nanoparticles to various breast cancer cell lines in vitro. Subsequently, the binding efficiency was also tested in mouse xenograft models. This approach is intended to guide surgeons during tumor resection by providing them with a tool to sensitively and specifically identify tumor margins while still in the OR, thus avoiding costly follow-up surgeries.

## 2. Materials and Methods

### 2.1. SERS Nanoparticles

**Figure 1:**
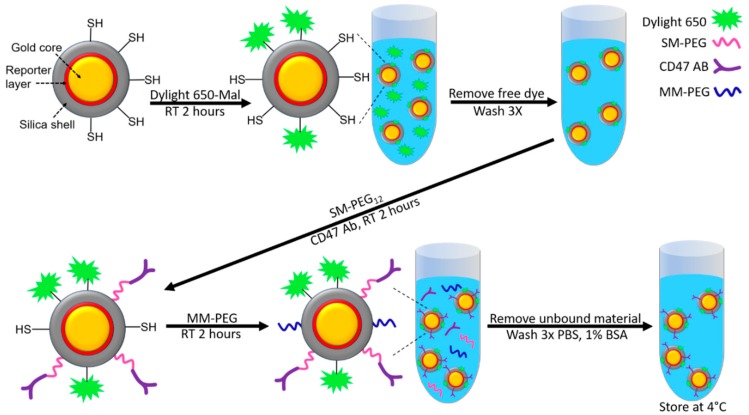
Schematic illustration of SERS NP conjugation with various elements, including Dylight 650 fluorophore, and PEGylated CD47 antibody.

The thiol functionalized NPs were obtained from Cabot Security Materials Inc., Mountain View, CA. The particles consist of a 60 nm gold core coated with a Raman reporter dye and encapsulated within a 30 nm silica shell (Figure 1). The Raman reporters used in this study included S440 (Trans-1,2-Bis(4-pyridyl)-ethylene) and S421 (d8-4,4′-dipyridyl) [2]. Extensive characterization, including transmission electron microscopy (TEM), dynamic light scattering (DLS), zeta potential, and energy-dispersive X-ray scattering (EDS), of these materials has been undertaken previously [1,2,21,22,23]. Additional DLS measurements provided here also correlate with the uncoated particle diameter of 140 ± 5 nm. After coating with the SM(PEG)_12_ and CD47 antibodies, we see an increase to a diameter of 150 ± 5 nm. Given the length of the polyethylene glycol (PEG) and antibody molecules, an increase in the hydrodynamic radius of ~10 nm is expected using DLS to measure these materials.

### 2.2. Conjugation of Fluorophore and CD47 Antibody to SERS Nanoparticle Surface

To ensure all the thiol groups on the surface of the NPs are available and reduced prior to conjugation, the NPs were briefly treated with Dithiothreitol (DTT). NPs in their storage buffer were centrifuged at 1500 *g* for 10 min, the storage buffer was removed, and the particles were resuspended in 10 mM DTT in 10 mM 3-(N-morpholino) propanesulfonic acid (MOPS) buffer at pH 7.4 and incubated at room temperature for 30 min. After incubation, the NPs were washed via centrifugation at 1500 G for 10 min and resuspended in MOPS buffer 5 times to ensure all the DTT had been removed.

The stock NPs were suspended at 400 pM in 10 mM in MOPS buffer at a pH of 7.4. As described in Figure 1, Dylight 650-maleimide (Thermo fisher #62295) was diluted in dimethylformamide (DMF) and mixed into the NP solution at a molar ratio of 60,000 dye molecules per nanoparticle and incubated at room temperature for 2 h. The resulting NP solution was then washed via centrifugation at 2000 G and resuspended in 10 mM MOPS 3 times.

Antibody conjugation (either anti-CD47 or IgG4K (isotype control)) was carried out using SM(PEG)_12_ (Sigma Aldrich, St. Louis, MO, USA #670278) as the crosslinker. The SM(PEG)_12_ was diluted to 0.25 mM with anhydrous DMF of which 5.12 µL was added to the NP solution giving a ratio of 3200 SM(PEG)_12_ per nanoparticle. The SM(PEG)_12_ was then added to a volume of the Dylight 650 conjugated SERS nanoparticles along with the specified antibody at a ratio of 200 per nanoparticle. This solution was then incubated at room temperature for 2 h (Figure 1). Following this conjugation reaction, methyl-terminated, polyethylene glycol with maleimide (MM-PEG) (Thermo Fisher, Waltham, MA, USA #22711) was added at a ratio of 600,000 per particle and incubated for 2 h at room temperature to saturate/protect any remaining unbound thiols (Figure 1). The resulting NP solution was then washed via centrifuge at 2000 G and resuspended in phosphate buffered saline (PBS) with 1% BSA three times before the final resuspension in the storage buffer, which consisted of PBS with 1% BSA at pH 7.4 and 0.03% sodium azide as preservative, and then stored at 4 °C (Figure 1). Dynamic light scattering indicated an increase in overall NP size of approximately 10 nm post conjugation of the CD47 antibody as described above.

### 2.3. Cell Culture and Flow Cytometry

Breast cancer cell lines listed in Table 1 were maintained using their respective culturing medias as suggested from the American Type Culture Collection (ATCC). The media was supplemented with 10% fetal bovine serum (FBS) and 1% Antibiotic/Antimycotic. Cells were cultured in an incubator at a temperature of 37 °C and 5% CO_2_. TrypLE express (Thermo Fisher Scientific, Waltham, MA, USA) was used to detach adherent cells for flow cytometry. In addition to the cell lines listed in Table 1, we used two separate DLD1 cell lines that were positive and negative for CD47 expression to act as our positive and negative controls, respectively. CD47 knock-out in DLD1 cells was achieved with a TALEN, and both cell lines were provided by D. Kurtz and I. Weissman. In preparation for flow cytometry, each cell line was diluted to a concentration of 2 million cells per mL. We tested 4 binding scenarios, including (1) control of cells alone, (2) cells with SERS-IgG NPs, (3) cells with SERS-CD47 NPs, and (4) cells with CD47 antibody (no NPs). The SERS NPs were conjugated with DyLight 650 to assess binding via FACS. The group given CD47 antibody alone was also conjugated with DyLight 650.

To assess binding of these various groups to each of our cell lines, 200,000 cells in 100 uL were placed in a microfuge tube. Then, 10 uL of our SERS-NPs at a concentration of 1.5 nM were incubated with the 100 uL of 200,000 cells in the microfuge tube in a cold room for 15 min. The reaction was quenched by adding 1 mL of PBS with 1% BSA. The cells were centrifuged at 150 *g* for 3–5 min to bring down the cells, but keep the unbound nanos in the supernatant. The supernatant was removed and the cell pellet was resuspended with 1 mL of PBS + 1% BSA to achieve a final volume of ~200,000 cells/mL for FACS. We used a Guava EasyCyte (Millipore Sigma, Burlington, MA, USA) flow cytometer, which has a convenient 96 well plate reader format. We plated 200 uL of our final volume to each well to achieve a cell count of ~40,000 cells per well. The fluorescence intensity for each of the cell populations counted was measured and analyzed using the GuavaSuite Guavasoft version 3.2 software package, (Millipore Sigma, Burlington, MA, USA).

### 2.4. Xenograft Tumor Models

Female 8-week-old nude mice (Charles River, Wilmington, MA, USA) were used for all tumor inoculation studies. All procedures performed on the animals were approved by the University’s Institutional Animal Care and Use Committee (APLAC# 30018/20786) and were within the guidelines of humane care of laboratory animals. Various breast cancer cell lines, as listed in Table 1, were cultured under standard conditions along with the positive and negative-expressing CD47 DLD1 cell lines. The xenograft tumor models were generated by subcutaneous injection of 5 million cells in 50 µL HBSS mixed with 50 uL of Matrigel on the flanks of the mice. Tumors were then harvested for SERS NP staining and Raman imaging from euthanized mice when the tumor volume reached approximately 200–300 mm^3^.

### 2.5. Human Tissue Specimen Acquisition

Deidentified human breast tissue specimens were obtained from consenting patients and imaged within 1 h after lumpectomy or mastectomy at the University of Washington Medical Center (informed consent was obtained from all patients). Tissue collection was managed by the Northwest BioTrust under an Institutional Review Board exemption for these deidentified tissues. The use of human specimens was approved by the University of Washington Institutional Review Board and was conducted in accordance with the provisions of the Declaration of Helsinki.

### 2.6. Tissue Staining with SERS NPs

Excised tissues were put on ice in preparation for staining. The tissues were cut in half to properly expose the flat tissue area to our NP stain. A mixture of S440-CD47 NPs (300 pM) and S421-Isotype NPs (300 pM) was used for staining the tissues. The isotype nanoparticles served as a control to assess non-specific binding. A glass slide was blotted with ~20 uL of this NP mixture for each tissue sample to be stained. The tissues were placed flat-side down in the NP mixture for a total of 5 min, during which they were briefly lifted and then re-dipped into the NP mixture at 20 s intervals to achieve optimal binding results, as described in a convection-enhanced topical staining protocol by Wang et al. [24]. After the 5 min of convection-enhanced staining, the tissues were rinsed in PBS for 20 s and placed upright on a quartz slide for Raman imaging.

### 2.7. Raman Imaging

The tissues were imaged using our home-built Raman endoscope system as described in Zavaleta et al. [1,25]. The SERS NP imaging system consists of a semiconductor diode near-infrared excitation laser operating at 785 nm with a laser power of approximately 42 mW measured at the surface of the tissues. The excitation beam is collimated to a ~1 mm spot size to maintain a consistent Raman signal as the beam is scanned across the heterogeneous tissue surface. Raman images were obtained by raster scanning the tissue of interest and creating intensity maps of respective nanoparticle concentrations. A computer-controlled x–y translation stage was used to raster-scan the tissues in order to obtain a laser-induced spectrum (primarily SERS and autofluorescence background) at each individual pixel within the area of interest with a 1 mm step size. The spectral integration time (each pixel) was 200 ms.

### 2.8. Raman Spectral Analysis

Images of NP concentrations (or concentration ratios) were generated by using either a principle component analysis (PCA) [26] or a direct classical least squares (DCLS) method, also known as the linear un-mixing and K-matrix methods [27,28]. DCLS finds the linear combination of spectra from the pure components within the sample that most closely matches the mixed spectrum acquired from each pixel of the sample. A component reference spectrum of the SERS nanoparticles used in this study was acquired for 1 s from a pure 5 µL mixed sample of S440-CD47 NPs and S421-Isotype NPs pipetted onto a piece of quartz under the microscope and used as the reference spectrum for the Raman analysis of the tissues harvested from the mice. Each CCD acquisition is saved and tagged with the respective x–y coordinate at which it was acquired. The acquisitions are then converted to a weighting factor using the DCLS algorithm and stored in a matrix, where each element of the matrix represents a pixel corresponding to the x–y coordinate at which the signal was acquired. The matrix of weighting factors (i.e., relative concentrations of the various NP “flavors” used in the staining mixtures) was then transformed into an image that is scaled to make use of the full 32-bit grayscale range. Finally, the Amide medical imaging data software was used to apply a Gaussian blur to the mapped image to give it smooth contours [29].

### 2.9. Statistics

The data collected from this study were analyzed for statistical significance using a 95% confidence interval (*p* < 0.05). A Student’s *t*-test was used to compare the Raman imaging data of the tumor groups to the data of the normal adjacent tissue groups. An equality of variances test was performed and revealed little variance between the groups. Therefore, a one-tailed *t*-test assuming equal variances was performed to determine statistical significance because it was hypothesized that the tumor groups would have more localized Raman signal from the NPs targeting CD47 on the breast cancer, whereas the normal adjacent tissue group would have less Raman signal due to less expression of CD47 and therefore less specific targeting. The values herein are reported as mean ± standard error of mean (SEM). The data from each of the time points correlated well with each other. Therefore, a Bonferroni correction was not indicated as it was too conservative, and there was little chance of getting a significant result from multiple *t*-testing.

## 3. Results

In this study, we first assessed the expression levels of CD47 in various breast cancer cell lines using FACS. We evaluated seven different breast cancer cell lines with varying expression levels of estrogen, progesterone, and HER2 (Table 1).

We also evaluated two DLD1 colon cancer cell lines to serve as our positive and negative controls. DLD1 is known to overexpress CD47, so we used this cell line to serve as our positive control and our negative control was derived from the same DLD1 cell line that underwent a TALEN-mediated knockout of CD47.

**Figure 2:**
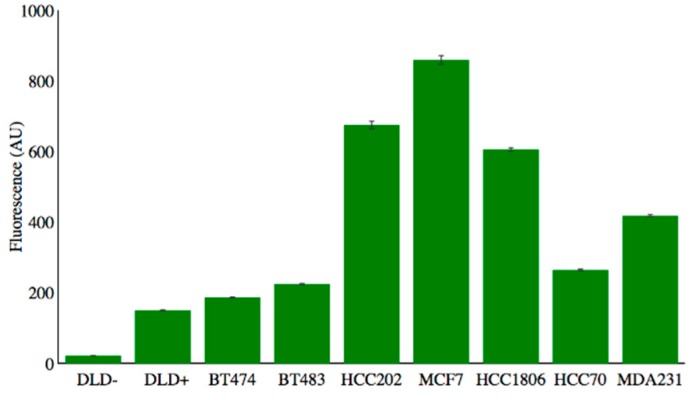
FACS data showing the binding efficiency of fluorescently labeled CD47 antibodies to various cell lines.

The various cell lines were incubated with a fluorescently labeled CD47 antibody to evaluate the expression levels of CD47 within each cell line. Figure 2 shows the binding efficiency of the CD47 antibody to the various cell lines tested. Note the negatively expressing cell line, DLD−, shows little expression of CD47 whereas all the breast cancer cell lines show overexpression of CD47 to varying degrees.

**Figure 3:**
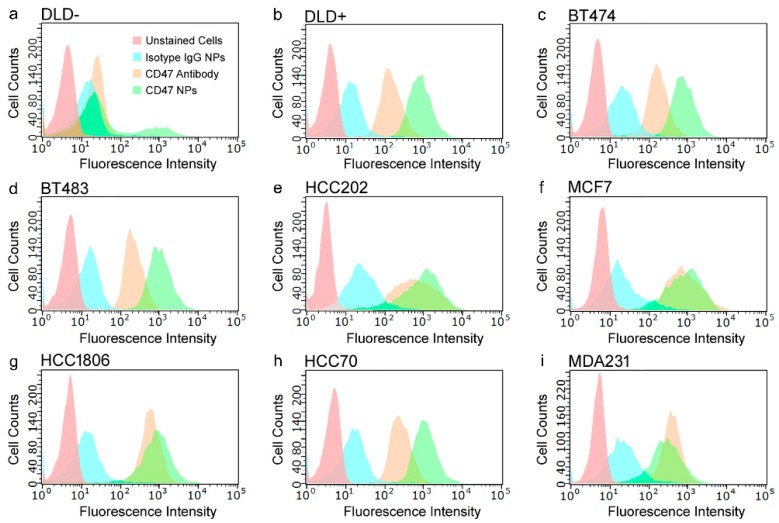
FACS plots showing the fluorescence intensity associated with each cell line tested including: (**a**) DLD−; (**b**) DLD+; (**c**) BT474; (**d**) BT483; (**e**) HCC202; (**f**) MCF7; (**g**) HCC1806, (**h**) HCC70; and (**i**) MDA231. The following experimental groups were tested within each of the cell lines (1) Unstained cells (**red**), (2) cells incubated with IgG SERS NPs (**blue**), (3) cells incubated with CD47 antibody (**orange**), and (4) cells incubated with CD47 SERS NPs (**green**). FACS was used to assess which cell lines demonstrated significant binding with our CD47 SERS NPs.

The CD47-targeted NPs were also evaluated using FACS to assess their binding efficiency to each of the cell lines described above. We had two distinct nanoparticle constructs, (1) SERS NPs (S440) conjugated to anti-CD47 mAb, and (2) SERS NPs (S421) conjugated to IgG, an isotype control antibody. Each of the nanoparticle constructs were labeled with the fluorophore, DyLight 650, as described in the materials and methods section. The unlabeled cell lines alone were also run through FACS to serve as our non-fluorescent control cell population. FACS revealed consistent binding of our SERS-CD47 NPs to each of the breast cancer cell lines as opposed to the negatively CD47 expressing cell line DLD− (Figure 3).

**Figure 4:**
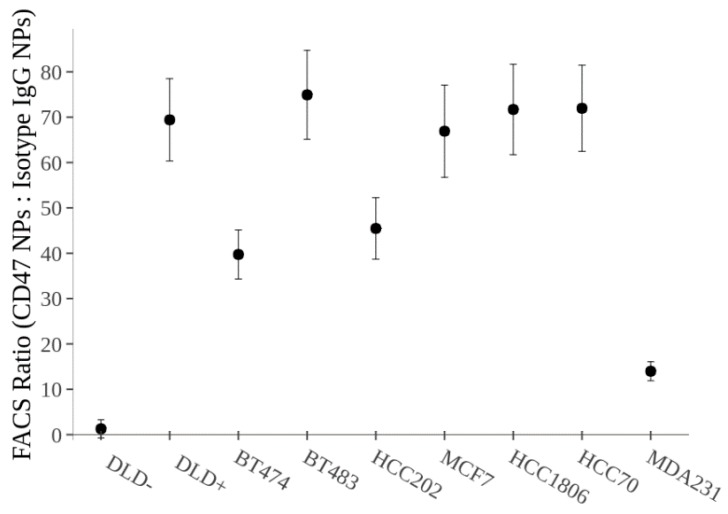
FACS data showing the ratio of CD47 labeled SERS NPs to Isotype IgG labeled SERS NPs. This data reveals the specific binding of our CD47 SERS NPs to each of the cell lines. The Isotype NPs are acting as a control to reveal non-specific binding of the NPs to the cell surface. Note the significant difference (*p* < 0.01) between the negatively CD47 expressing cell line, DLD−, and the rest of the cell lines.

As shown in Figure 4, the isotype IgG-labeled SERS NPs showed significantly less binding (*p* < 0.01) in the breast cancer cell lines than CD47-targeted SERS NPs. Since this isotype NP construct was labeled onto a different “flavor” of SERS NP (S421), we will be able to administer a mixture of both the CD47 labeled SERS NP (S440) and the Isotype IgG labeled SERS NP (S421) simultaneously and perform ratiometric imaging to account for non-specific binding of the NPs. This unique multiplexing strategy allows for real-time evaluation of the specific vs. nonspecific binding of NPs to tissues (tumor and benign) and is valuable for tumor margin imaging applications in which a large number of nonspecific (and misleading) sources of contrast can arise [30,31]. Figure 4 shows the ratio of CD47 labeled SERS NPs to isotype IgG labeled SERS NPs. Notice how all the breast cancer cell lines show significant binding with our CD47 labeled SERS NPs, apart from our negatively expressing CD47 cell line, DLD−.

**Figure 5:**
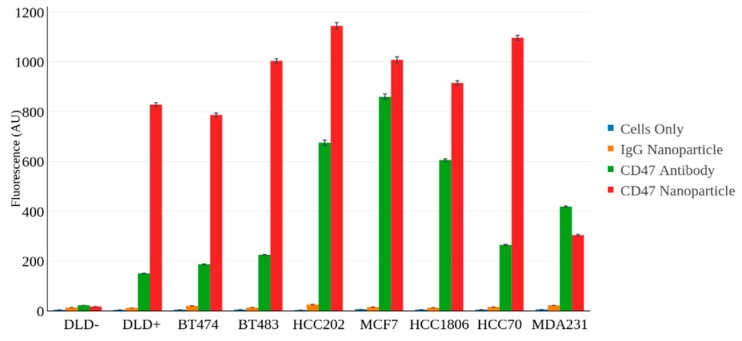
Quantitative FACS data comparing signal from cells only (**blue**), cells labeled with an isotype-NP (**orange**), cells labeled with anti-CD47 mAb (**green**), and cells labeled with CD47-NP (**red**).

For a more comprehensive and quantitative assessment of all the FACS runs performed in this study refer to Figure 5. Notice how the overexpression of CD47 antibody correlates well with the CD47 SERS NP binding. In most cases, there appears to be more fluorescence signal associated with the cells incubated with the CD47 NPs as opposed to the cells incubated with the CD47 antibody alone. This could be explained by the fact that the binding event of one NP with several bound fluorophores will likely “outshine” the binding event of one CD47 antibody bound with a single fluorophore. The multi-valent binding potential that the hundreds of antibodies on a single NP have over single antibodies could also play a significant role in the increased binding behavior observed. These FACS results clearly support the idea of utilizing CD47 SERS NPs as imaging contrast agents in these breast cancer cell lines.

**Figure 6:**
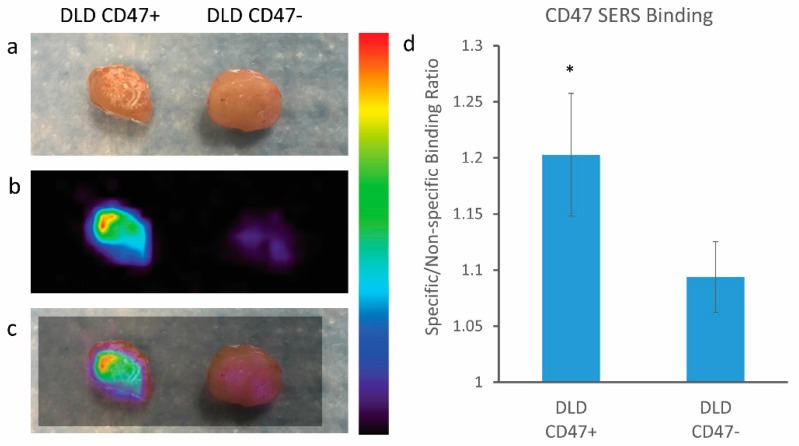
Raman imaging of positive and negative CD47 expressing tissues (DLD+ and DLD−, respectively) harvested from mouse xenograft. (**a**) Digital photo of excised tissue after NPs administration; (**b**) Raman imaging of tissue samples; (**c**) overlay of Raman imaging with tissue sample, notice the increased NP binding in the tissue expressing CD47 as opposed to the negatively CD47 expressing tissue; (**d**) quantitative ratiometric analysis of specific CD47 SERS NP binding to non-specific Isotype IgG SERS NP binding on tissue samples Notice the significant differences represented by * (*p* < 0.05) between the positive control (DLD CD47+) and the negative control (DLD CD47−) tissues; error bars represent standard error of mean (SEM). Color bar to the right of Raman images represents Raman intensity, where red represents the highest Raman signal and black represents no associated Raman signal.

Moving forward, we chose to assess the binding potential of our CD47 SERS NPs on fresh tissue samples. We grew subcutaneous tumor xenografts in mice and then excised the tumors to evaluate the binding efficiency of CD47 SERS NPs on fresh tissue samples excised from the mice. Our first tissue study looked at the binding potential of our SERS NPs on the positive and negative control cell lines, DLD+ and DLD−, respectively. The cells were grown on the flanks of mice to a tumor size of ~300 mm^3^. The mouse was euthanized, and the tumors were harvested for SERS NP administration. The tumors were cut in half to expose fresh tissue to a mixture of CD47 SERS NPs (S440) and IgG SERS NPs (S421). The respective tissues (DLD+ and DLD−) were dipped in a solution of NPs for 5 min as described in Section 2.4 of the Materials and Methods. The unbound NPs were then rinsed away from the tissue with PBS for 20 s. The tissues were then imaged with our home-built Raman system to reveal specific binding of the NPs. Figure 6 reveals significant binding of our CD47 SERS NPs to the DLD+ tissue overexpressing CD47 as opposed to the negative control tissue (DLD−). This data suggests that Raman imaging can detect the presence of positively expressing CD47 on a freshly excised tissue sample.

**Figure 7:**
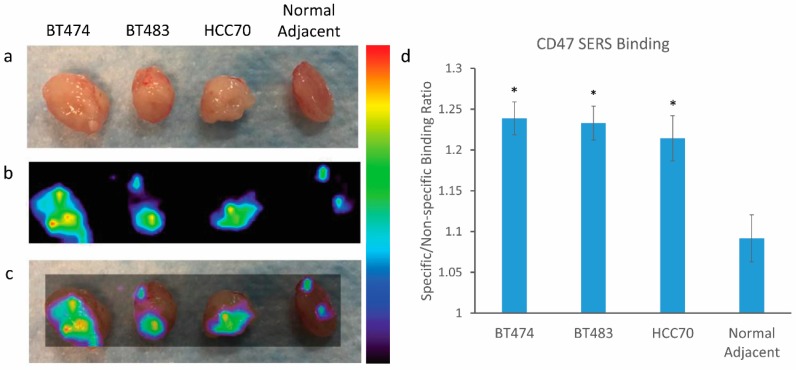
Raman imaging of breast cancer tissues and normal adjacent tissue harvested from mouse xenograft. (**a**) Digital photo of excised tissue after NPs administration; (**b**) Raman imaging of tissue samples; (**c**) overlay of Raman imaging with tissue sample, notice the increased NP binding in the breast cancer tissue as opposed to the normal adjacent tissue; (**d**) quantitative ratiometric analysis of specific CD47 SERS NP binding to non-specific Isotype IgG SERS NP binding on each of the tissue samples. Notice the significant differences represented by * (*p* < 0.05) between the cancer tissue and the normal adjacent tissues; error bars represent standard error of mean (SEM). Color bar to the right of Raman images represents Raman intensity, where red represents the highest Raman signal and black represents no associated Raman signal.

Next, we assessed the tumor targeting ability of our Raman imaging approach on multiple breast cancer tissues. We chose to focus on three distinct breast cancer cell lines: BT474, BT483, and HCC70. Each of these cell lines had varying expression characteristics of estrogen receptor, progesterone receptor, and HER2, including the triple negative cell line, HCC70 (Table 1). The cells were grown on the flanks of mice to a tumor size of ~300 mm^3^. The mouse was euthanized, and the tumors as well as the normal adjacent tissues were harvested for SERS NP administration as described above. The tissues were rinsed and then imaged with our Raman imaging system to reveal specific binding of the NPs. Figure 7 reveals significant binding of our CD47 SERS NPs to all three breast tissue cell lines as opposed to the normal adjacent tissue taken from each of the mice. This data suggests that Raman imaging can detect the difference between the positively expressing CD47 breast cancer tissue and the neighboring normal tissue surrounding it. Note that the difference in expression levels of estrogen receptor, progesterone receptor, and HER2 between the different cell lines does not significantly affect the ability of our approach to detect the breast cancer. This is an important feature of this approach since it does not exclude triple negative breast cancer patients from its potential benefits. These results strongly support this Raman imaging approach for image-guided surgery during lumpectomy.

## 4. Discussion

Breast cancer continues to rank among the most common cancers in women, affecting one in eight women in the United States [32]. Currently, roughly one in four women who have a lumpectomy must undergo additional surgery, which indicates the importance of improved surgical precision [33]. Our results suggest that Raman imaging of our CD47 targeting SERS nanoparticles could play a significant role in identifying and localizing residual breast cancer to help guide surgeons in the OR. As outlined in Table 1, we assessed seven breast cancer cell lines, each expressing varying combinations of the three main biomarkers (ER, PR, HER2) used to clinically manage breast cancer patients. Our results showed that all the breast cancer cell lines overexpressed CD47 to a significant degree regardless of their expression of ER, PR, or HER2.

More recently, image guided surgery has generated quite a bit of interest in the field of molecular imaging, with several investigators assessing various contrast agents along with various imaging techniques [34,35,36,37,38,39,40,41,42,43]. Both preclinical and clinical studies have shown the benefits that image guidance can offer during all sorts of surgical applications. Wang et al. recently reported the use of near-infrared II fluorescent emitting nanoparticles modified with tumor targeting peptides to improve surgical resection of metastatic ovarian cancer in preclinical models [43]. Their technique was able to identify and remove metastases ≤1 mm with better image quality and deeper tissue penetration than that of clinically approved small molecule indocyanine green (ICG). Gao et al. reported on an entirely different application for utilizing fluorescent imaging to guide head and neck squamous cell carcinoma surgery [36]. They evaluated their strategy on 21 adult patients scheduled for standard of care surgery. Patients received a tumor targeting fluorescent dye, panitumumab-IRDye800CW, via intravenous (IV) administration. Images revealed three-fold signal difference between positive and negative specimens with a high correlation of fluorescent signal associated with the tumor’s location revealing high sensitivity and specificity >89%. These results suggest that fluorescence imaging can be used to guide head and neck surgeries with improved resection and patient outcome. Another group investigated the use of image guidance during video-assisted thoraoscopic surgery for the resection of pulmonary nodules [44]. ICG dye was administered to patients percutaneously under cone beam computed tomography to mark/localize the tumor. Real-time fluorescence imaging was then performed to guide subsequent resection. All pulmonary lesions were identified via NIR imaging, with no adverse events supporting that this strategy can successfully localize non-visible, non-palpable pulmonary nodules. More recently, the food and drug administration (FDA) approved aminolevulinic acid hydrochloride, known as ALA HCl, as an imaging agent for patients with gliomas to aid in visualization of malignant brain tissue during surgery. This imaging agent elicits accumulation of fluorescent protoporphyrin IX (PPIX) in malignant glioma tissue and is used for fluorescence-guided tumor resections. Several clinical trials supported its efficacy, revealing a more “complete” resection of tumor in patients as compared to the control arm [45,46].

The added advantages that Raman imaging with SERS nanoparticles has to offer over other conventional approaches, like fluorescence imaging, include its ultrahigh sensitivity and multiplexing capabilities [2,22]. Our group has previously reported that Raman imaging with SERS NPs is ~1000 times more sensitive than the fluorescence imaging equivalent with quantum dot NPs [2]. Raman imaging, being an optical-based technique, is an ideal modality for surface imaging on fresh tissues as it is relatively fast and non-destructive. In the future, it may be possible to topically apply a cocktail of various tumor targeting SERS nanoparticles each targeting various breast cancer biomarkers simultaneously. This approach would greatly increase the imaging specificity of breast cancer. Wang et al. have demonstrated the use of a similar Raman imaging strategy for the detection of four biomarkers: EGFR, HER2, CD44, and estrogen receptor (ER) [31,47]. As our understanding of breast cancer biomarkers continues to improve, we hypothesize that multiplexed molecular imaging of breast cancer will improve the completeness of resection and thus improve breast cancer patient outcomes.

The addition of CD47 to this SERS nanoparticle cocktail could have beneficial effects, especially in cases where breast cancer patients do not express HER2 or ER. Around 20% of breast cancer patients have triple negative breast cancer [48], in which their tumors do not express ER, PR, or HER2. Since these women are not eligible for certain hormone therapies, complete surgical resection is even more important [49]. African American and Hispanic women have a higher incidence of triple negative breast cancer and poorer survival rates [50], representing a subpopulation of patients in need of alternative biomarkers. As validated in this study, CD47 has shown high levels of expression on breast cancer cells, including triple negative breast carcinoma cells, and could serve as a valuable biomarker to target for both imaging and therapy [51].

CD47 has already been investigated as a biomarker for therapy, so its promise as an imaging target is well justified. As a result of the overexpression observed on several solid tumors, researchers have begun to investigate the potential of CD47 as a diagnostic imaging target. Ying Pan et al. showed the potential of using a CD47 antibody to image human bladder cancer with fluorescence imaging [52]. They used fluorescent quantum dots labeled with CD47 antibodies, which were topically delivered to fresh intact resected bladder specimens. They reported an 82.9% sensitivity and 90.5% specificity for CD47-targeted imaging of bladder cancer with their fluorescent imaging approach, suggesting its potential for improving cancer detection and enabling image-guided surgery. The same group recently reported on the use of CD47 tumor targeting SERS nanoparticles using Raman imaging for the detection of bladder cancer and found similar results supporting the use of CD47 as a diagnostic imaging target to potentially guide transurethral surgery [53]. They also observed a passive accumulation of SERS nanoparticles to the bladder cancer that they describe as an enhanced surface permeability and retention effect in human bladder cancer [53].

**Figure 8:**
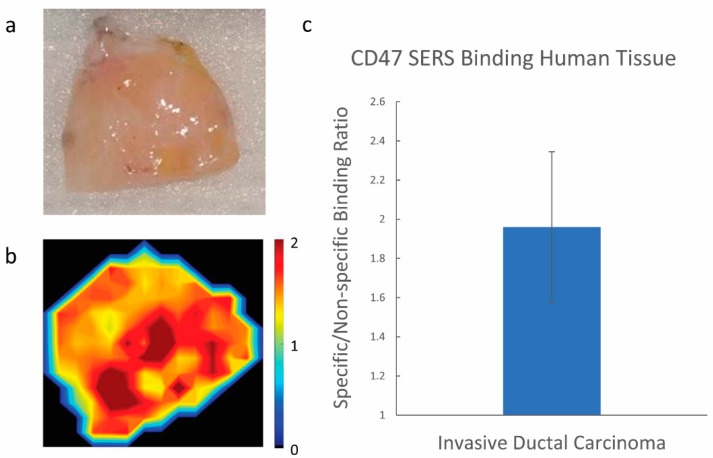
Raman imaging of resected invasive ductal carcinoma specimen from a 55-year-old patient. (**a**) Digital photo of excised tissue after NPs administration; (**b**) Raman imaging of tissue sample; (**c**) quantitative ratiometric analysis of specific CD47 SERS NP binding to non-specific Isotype IgG SERS NP binding on the human tissue sample. Scale bar represents the specific/non-specific binding ratio of our targeted/isotype-labeled nanoparticles.

Nanoparticles have had a challenging time translating to the clinic, especially in the context of imaging applications. This is often due to their prolonged retention and potential systemic toxicity post IV administration. As a result, several researchers are looking toward utilizing topical approaches to avoid negative systemic delivery effects [24,31,47,52,53,54,55,56,57,58]. Our topical approach of administering nanoparticles directly to the resected tissue, investigated here, supports the idea that our tumor targeting nanoparticles can still provide both sensitive and specific information between tumor and normal adjacent tissue specimens post resection. This approach, investigated here, in turn eliminates any concern regarding systemic toxicity since there is no direct administration of nanoparticles to the patient. As an example, we were able to generate some preliminary data to show the potential this strategy has for targeting breast cancer in a human breast cancer specimen resected from a 55-year-old patient with invasive ductal carcinoma. Notice the specific binding of our CD47 targeted nanoparticles to the breast cancer specimen as opposed to our isotype labeled nanoparticles (Figure 8). This preliminary data supports further investigation of this nano-based imaging approach on clinical human samples with normal adjacent tissue margins to evaluate its potential in guiding surgical resection.

Notably, our proposed approach is not limited to breast cancer surgery, but generalizable to other solid cancers where organ-sparing surgery is paramount (e.g., head and neck cancers, melanoma, pancreatic cancer, brain cancer, and kidney cancer). As previously mentioned, CD47 has been shown to be overexpressed in several tumor types, including ovarian, colon, stomach, bladder, glioblastoma, hepatocellular carcinoma, and prostate cancers [20]. Our CD47 tumor targeting SERS nanoparticles could be applied topically to a number of these tissues during intraoperative tumor resection. Alternatively, we recently reported that oral delivery of our nanoparticles reveals no evidence of systemic toxicity and complete clearance from the body via the gastrointestinal (GI) tract within 24 h post ingestion [21]. This approach could be particularly useful in areas along the GI tract that overexpress CD47, like the stomach and colon, when used with a Raman endoscopic imaging device [1,25,30,47]. This approach could provide endoscopists and surgeons with a molecular map that offers real-time information to either sensitively diagnose or resect cancer.

## 5. Conclusions

In summary, all seven of the breast cancer cell lines we investigated, including three triple-negative lines, showed overexpression of CD47. Consequently, we investigated the tumor targeting and imaging potential of CD47-targeted SERS nanoparticles in cells and on excised preclinical tissue samples. Raman images revealed effective targeting of our SERS nanoparticles to positively expressing CD47 breast tumors as opposed to normal adjacent tissue. Despite the toxicity concerns associated with nanoparticles, their many advantages over small molecule contrast agents should encourage further investigation for clinical translation. This study clearly shows a non-toxic path forward for utilizing these ultrasensitive SERS nanoparticles in guiding surgical resection with Raman imaging, and is an important first step towards their clinical translation. In conclusion, the imaging strategy presented here successfully distinguished tumor vs normal adjacent tissue in preclinical models and should be further investigated in clinical human samples to assess its potential role in guiding surgical resection.

## Figures and Tables

**Table 1:** Breast cancer cell lines interrogated for CD47 expression.

Cell Line	Tumor Type	Estrogen	Progesterone	HER2
BT474	Primary, IDC *	+	+	+
BT483	Primary, IDC *	+	+	−
HCC202	Primary, DC *	−	−	+
MCF7	Metastatic, Pulmonary Effusion	+	+	−
HCC1806	Primary, Squamous Carcinoma	−	−	−
HCC70	Primary, DC *	−	−	−
MDA231	Adenocarcinoma	−	−	−

* DC: Ductal Carcinoma, IDC: Invasive Ductal Carcinoma.
